# A Machine Learning Approach for Identifying People With Neuroinfectious Diseases in Electronic Health Records: Algorithm Development and Validation

**DOI:** 10.2196/63157

**Published:** 2025-08-29

**Authors:** Arjun Singh, Shadi Sartipi, Haoqi Sun, Rebecca Milde, Niels Turley, Carson Quinn, G Kyle Harrold, Rebecca L Gillani, Sarah E Turbett, Sudeshna Das, Sahar Zafar, Marta Fernandes, M Brandon Westover, Shibani S Mukerji

**Affiliations:** 1 Department of Neurology, Massachusetts General Hospital, 55 Fruit St, Wang ACC 835, Boston, MA, 02114, United States, 1 2163379887; 2 Harvard Medical School, Boston, MA, United States; 3 Department of Neurology, Beth Israel Deaconess Medical Center, Boston, MA, United States; 4 Department of Neurology, Brigham and Women’s Hospital, Boston, MA, United States; 5 Department of Pathology, Massachusetts General Hospital, Boston, MA, United States; 6 Department of Medicine, Massachusetts General Hospital, Boston, MA, United States

**Keywords:** neurology, neuroinfectious disease, electronic health records, EHR, machine learning, artificial intelligence, neuroinfectious, develop, validate, expressions, logistic regression, clinical notes, efficiency

## Abstract

**Background:**

Identifying neuroinfectious disease (NID) cases using International Classification of Diseases billing codes is often imprecise, while manual chart reviews are labor-intensive. Machine learning models can leverage unstructured electronic health records to detect subtle NID indicators, process large data volumes efficiently, and reduce misclassification. While accurate NID classification is needed for research and clinical decision support, using unstructured notes for this purpose remains underexplored.

**Objective:**

The objective of this study is to develop and validate a machine learning model to identify NIDs from unstructured patient notes.

**Methods:**

Clinical notes from patients who had undergone lumbar puncture were obtained using the electronic health record of an academic hospital network (Mass General Brigham [MGB]), with half associated with NID-related diagnostic codes. Ground truth was established by chart review with 6 NID-expert physicians. NID keywords were generated with regular expressions, and extracted texts were converted into bag-of-words representations using n-grams (n=1, 2, 3). Notes were randomly split into training (80%), 2400 notes out of 3000, and hold-out testing (20%), 600 notes out of 3000, sets. Feature selection was performed using logistic regression with L1 regularization. An extreme gradient boosting (XGBoost) model classified NID cases, and performance was evaluated using the area under the receiver operating curve (AUROC) and the precision-recall curve (AUPRC). The performance of the natural language processing (NLP) model was contrasted with the Llama 3.2 auto-regressive model on the MGB test set. The NLP model was additionally validated on external data from an independent hospital (Beth Israel Deaconess Medical Center [BIDMC]).

**Results:**

This study included 3000 patient notes from MGB from January 22, 2010, to September 21, 2023. Of 1284 initial n-gram features, 342 were selected, with the most significant features being “meningitis,” “ventriculitis,” and “meningoencephalitis.” The XGBoost model achieved an AUROC of 0.98 (95% CI 0.96‐0.99) and AUPRC of 0.89 (95% CI 0.83‐0.94) on MGB test data. In comparison, NID identification using International Classification of Diseases billing codes showed high sensitivity (0.97) but poor specificity (0.59), overestimating NID cases. Llama 3.2 improved specificity (0.94) but had low sensitivity (0.64) and an AUROC of 0.80. In contrast, our NLP model balanced specificity (0.96) and sensitivity (0.84), outperforming both methods in accuracy and reliability on MGB data. When tested on external data from BIDMC, the NLP model maintained an AUROC of 0.98 (95% CI 0.96‐0.99), with an AUPRC of 0.78 (95% CI 0.66‐0.89).

**Conclusions:**

The NLP model accurately identifies NID cases from clinical notes. Validated across 2 independent hospital datasets, the model demonstrates feasibility for large-scale NID research and cohort generation. With further external validation, our results could be more generalizable to other institutions.

## Introduction

Meningitis and encephalitis pose serious threats to the health of an individual, potentially leading to severe neurological compromise or even death [1-7]. Many neuroinvasive pathogens, including viruses (eg, enterovirus, herpes simplex virus, West Nile virus, and HIV), bacteria (eg, *S. pneumoniae*, Mycobacterium tuberculosis), and fungi (eg, Cryptococcus), are linked to long-term cognitive sequelae [2,8]. Recent population-based studies suggest associations between pathogen exposures, especially neuroinvasive viruses causing encephalitis or meningitis, and the subsequent risk of developing dementia such as Alzheimer disease (AD) [9-12]. The risk of AD appears to increase with longer time since the first infection, peaking after 12‐30 years [9]. Prior viral encephalitis and bacterial or viral meningitis were found to be associated with significantly increased hazard ratios for future AD, with rates of 30.72-fold and 2.81-fold, respectively, based on the Finnish Biobank, suggesting that exposure to neuroinvasive pathogens may contribute to lower cognitive reserve, likely in part due to brain inflammation [10].

These findings underscore the need for comprehensive, longitudinal studies to elucidate the mechanisms by which neuroinvasive pathogens might contribute to neurodegenerative diseases. However, such research is severely hampered by the scarcity of large, well-annotated hospital datasets. This data gap not only impedes rigorous epidemiological analyses but also hinders mechanistic investigations that could reveal how pathogens interact with neural tissues to potentially trigger or accelerate neurodegenerative processes. Therefore, to support evidence-based clinical decision-making and enable large-scale, high-powered research into the long-term consequences of neuroinfectious diseases (NIDs), accurately identifying these conditions in hospital patient records remains a significant challenge.

NIDs stem from a diverse array of pathogens and often present with clinically indistinguishable symptoms such as fever, headache, and confusion. Physicians often grapple with challenges in promptly identifying these pathogens, resorting to less precise shotgun testing methods, leading to delayed or unconfirmed diagnoses, unnecessary antimicrobial treatments, and preventable morbidity [13-18]. Most epidemiological studies linking infectious disease burden to subsequent dementia rely on health care databases that use International Classification of Diseases (ICD) codes 9th (ICD-9) and 10th (ICD-10) revisions (combined as ICD codes) and do not validate diagnoses with microbial assays [19]. The limited reliability of ICD codes in accurately identifying infectious diseases has been previously reported [20-23], more recently underscored by the 56%‐57% positive predictive value observed in the Danish National Patient Registry investigating herpes simplex virus encephalitis [19]. The combination of ICD miscoding and the potential misclassification of encephalitis etiology poses significant obstacles to generating actionable recommendations for preventing AD and other dementias. This challenge further hinders clinical guidance, particularly in cases of viral encephalitis with unknown origins—a subgroup identified in epidemiological studies as having the highest risk of future dementia [10,11].

Recent efforts to move beyond ICD-based coding have aimed at improving the timely and accurate diagnosis of NID etiologies, particularly in differentiating between viral and bacterial meningitis. These efforts predominantly use approaches such as decision trees and ensemble methods. For example, a study using a Brazilian dataset of 12,774 patients described by 19 clinical attributes aimed at distinguishing bacterial from aseptic meningitis found that a hybrid bagging approach with Naive Bayes Trees outperformed 27 other tested models. This model achieved a sensitivity of 96% and a specificity of 82% [24]. While promising, most other classification studies on NIDs, using techniques like logistic regression [25-27] or hybrid models [24,27-32], are primarily in pediatric populations, lack external validation, or rely on small datasets with limited cross-validation [33]. To our knowledge, no study has used unstructured data such as clinical notes, which limits the insights that could be derived.

The 2015 global research priorities for infections that affect the nervous system underscored the need for accurate disease burden estimates and improved tools for neurological and cognitive impairment assessment [2]. Natural language processing (NLP) tools can leverage the rich information present in large-scale comprehensive electronic health records (EHRs), including unstructured clinical assessments and notes [34-36]. Therefore, NLP holds the potential to surpass the accuracy of traditional ICD-based phenotyping while reducing reliance on semi-structured data formats [30,35,37,38].

When structured lab data is unavailable, NLP techniques leveraging unstructured clinical notes can potentially generate more accurate NID cohorts than ICD codes alone. Many NLP algorithms are computationally lighter and require less computing infrastructure compared to more recent large language models (LLMs), making them more feasible for widespread research applications. Building on this advantage, our goal is to develop a more accurate and efficient method for classifying NID cases in EHRs. Previous studies have successfully used NLP to identify patients with epilepsy [38] or classify neurological outcomes from medical notes [39].

Here, we aim to tackle NID patients by building upon these studies and detailing our NLP algorithm leveraging discharge summaries and progress notes to identify NID patients and investigate the model’s accuracy across 2 independent hospitals. Our NLP algorithm outperforms ICD codes in identifying NID patients and achieves competitive performance compared to the Llama 3.2 autoregressive model (an LLM with 3B parameters) in zero-shot learning tasks, making it a valuable tool for large-scale EHR-based research to investigate the relationship between NID exposure and short- and long-term neurological outcomes.

## Methods

### Overview

**Figure 1.**
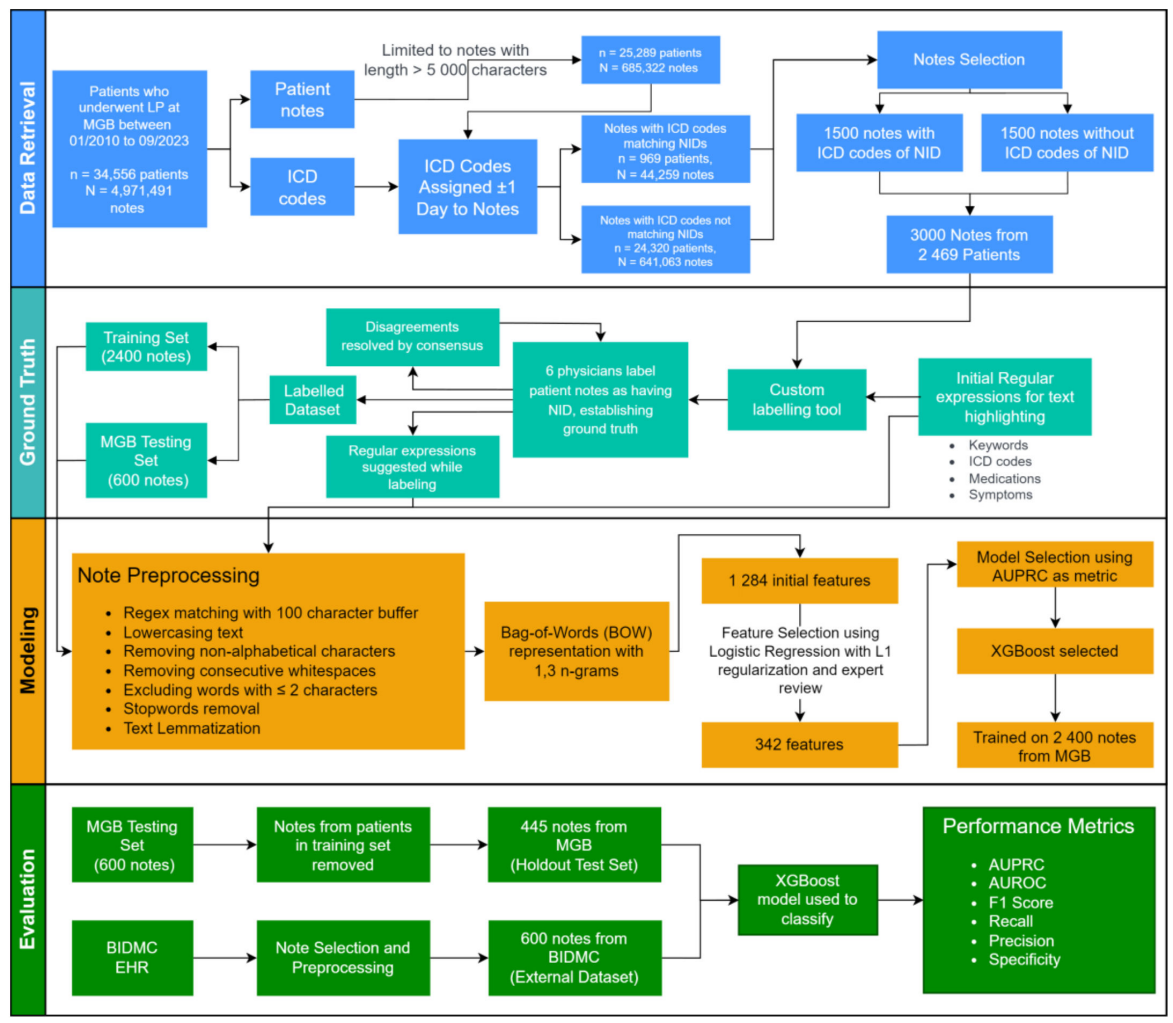
Methods overview: data retrieval and machine learning pipeline for NID classification. AUPRC: area under the precision-recall curve; AUROC: area under the receiver operating characteristic curve; BIDMC: Beth Israel Deaconess Medical Center; BOW: bag-of-words; EHR: electronic health record; ICD: International Classification of Diseases; LP: lumbar puncture; n: number of unique patients; N: number of unique notes; MGB: Mass General Brigham; NID: neuroinfectious diseases.

Figure 1 provides a summary overview of the key steps involved in this study, including data extraction, note selection, regular expression development, ground-truth labeling, text preprocessing, feature extraction, model training, and evaluation.

### Data Extraction and Note Selection

We included 34,556 patients who underwent lumbar punctures (LP), sourcing medical notes from the EHR database of the Mass General Brigham (MGB) network of hospitals. This approach enriched individuals likely to be evaluated for central nervous system (CNS) infections, with a total of 4,971,491 notes extracted between 01/22/2010 and 09/21/2023. ICD diagnostic codes billed one calendar day before and after the notes were extracted and labeled as likely to be associated with NID. Multimedia Appendix 1 lists the NIDX ICD 9 and 10 codes.

We excluded notes with fewer than 5000 characters, as they likely lacked the necessary detail for clinicians to accurately assess patient status or provide valid training for models. We categorized notes into two groups: (1) notes with NID-related ICD codes (969 unique patients, 44,259 notes) and (2) those without NID ICD codes (24,320 unique patients, 641,063 notes). From these 2 groups, we randomly selected 3000 notes from 2469 patients at MGB, of which 50%, 1500 out of 3000, were associated with NID-related ICD codes, and 50% were not. For external validation, we also extracted 600 inpatient notes from patients in the Beth Israel Deaconess Medical Center (BIDMC) EHR system located in the Brain Data Science Platform (bdsp.io) maintaining a 50:50 split of cases with and without NID-related ICD codes to match the MGB test set size.

### Ethical Considerations

All procedures involving human participants in this study were reviewed and approved by the Institutional Review Board (IRB) of MGB, under protocol #2013P001024. The research was conducted in accordance with the ethical standards of the MGB and the principles of the WMA Declaration of Helsinki.

This study involved analysis of EHR data. The IRB approved a waiver of informed consent due to the retrospective nature of the study and minimal risk to participants. In accordance with the IRB’s requirements, no identifiable personal information is included in the manuscript or supplementary materials. While the data were not fully de-identified at the time of analysis, strict procedures for maintaining privacy and confidentiality were implemented. Access to identifiable information was restricted to certified study personnel who have completed training in ethics and responsible conduct of research. The research team was committed to upholding high standards of data privacy and confidentiality throughout the entire study process. No compensation was provided to participants, as only de-identified retrospective data were utilized.

### Ground-Truth Labeling

In total, 39,245 regular expressions were formulated based on domain-specific knowledge, categorized as (1) positive NID (eg, “cerebrospinal fluid (CSF) positive for Epstein-Barr Virus via polymerase chain reaction,” “ID felt encephalitis and seizures triggered by a viral illness,” “Currently on empiric antibiotics and acyclovir for possible CNS infection”); (2) negative NID (eg, “LPs have been negative,” “no signs of any infectious,” “autoimmune epilepsy/encephalitis”); (3) NID drugs (eg, ceftriaxone, acyclovir, trimethoprim-sulfamethoxazole, etc); (4) NID-likely keywords (eg, “headache,” “fever,” “lumbar puncture,” etc). Regular expressions were developed by listing relevant organisms based on source materials for infectious diagnoses, antimicrobial agents, and symptoms associated with meningitis and encephalitis. Many terms were combined to create comprehensive positive and negative expressions. In addition to domain expert input, source materials included national guidelines related to CNS diseases in adults and pathogen data from global burden of disease studies [4,40-43]. The positive, negative, and antimicrobial regular expressions are provided in MultimediaAppendices 2^4^.

In total, 6 physicians (AS, CQ, KH, GH, ST, and SM), all domain experts in neuroimmunology or infectious diseases, independently classified 500 notes each from the MGB dataset (3000 notes in total) following a standardized operating procedure. This procedure provided detailed guidelines for reviewing notes, including assessing symptoms and evaluating laboratory tests when referenced in the notes. Ambiguous cases flagged by the physicians were reviewed by an independent physician who was not involved in the initial classification. A consensus approach was used to resolve discrepancies in classification. To facilitate labeling, a labeling software tool was created to show the notes while highlighting the keywords (eg, meningitis, encephalitis, ceftriaxone, etc) and phrases (eg, “meningitis, likely bacterial,” “CSF pleocytosis noted”*)*. Domain experts had the option to recommend additional regular expressions during the MGB ground-truth labeling phase. Regular expressions proposed by domain experts were incorporated if proposed on the training dataset only. External model validation on 600 BIDMC notes was classified by 2 experts (AS and SM) (300 notes each). While the MGB test set was reduced to 445 notes to address overlap with the training set, the BIDMC dataset was solely used for external validation and retained its full size of 600 notes.

### Preparing Text Data for Feature Extraction

Preprocessing notes to identify model features required (1) limiting text to regions matching regular expressions with a 100-character buffer, (2) converting to lowercase letters, (3) removal of non-alphabetical characters, (4) replacing consecutive whitespaces with a single whitespace, (5) using words with more than 2 characters, and (6) removing stop words (see Multimedia Appendix 5). The remaining text was lemmatized using WordNetLemmatizer [44]. The text was transformed into a bag-of-words (BOW) representation, considering unigrams (1 gram), bigrams (2 grams), and trigrams (3 grams).

### Model Features, Training, and Performance Evaluation

To reduce the number of features and enhance model interpretability, we used an iterative approach using logistic regression with L1 regularization, similar to our previous NLP-based methods on unstructured EHR text in studies on epilepsy or neurological status following COVID-19 hospitalizations [38,45]. The regularization strength (10) and maximum iterations (1000) were chosen to balance between model complexity and computational efficiency. We addressed class imbalances by incorporating class weights inversely proportional to the sample size. We used this process to identify the most common features, resulting in a set of 1284 features. A manual review by 3 experts (AS, SM, and MBW) eliminated the remaining irrelevant features, leading to a reduced set of 342 non-zero features. For example, terms like “Boston,” “heart rate,” and “primary care” were removed as they were irrelevant to NID classification and had no pathophysiological connection to the condition.

The MGB dataset (3000 notes) was randomly split 80:20 for training (2400 notes) and the hold-out (600 notes) test set, maintaining the 50/50 ICD code distribution. Since there were only 969 unique patients with ICD codes for NIDs, some notes originated from the same patients. To ensure no patient appeared in both the training and test sets, the initial hold-out test set of 600 notes was reduced to 445 notes by excluding notes from patients who were also present in the training set. To select the optimal model, we used 5-fold cross-validation on the MGB training dataset. Given the imbalanced nature of the dataset, with only 16%, 480 out of 3000, of the total observations positive for NID, we compared logistic regression, random forest, and XGBoost using the area under the precision-recall curve (AUPRC). AUPRC is more informative for imbalanced datasets, as it focuses on the performance of the model in predicting the positive class, unlike metrics such as accuracy or the area under the receiver operating characteristic curve (AUROC) [46-49]. XGBoost was selected for its AUPRC performance.

We then trained an XGBoost model on the 2400 training notes and tested its performance on both the hold-out dataset (445 notes from MGB) and an external dataset consisting of 600 notes from patients at BIDMC. Model performance was based on the AUPRC and the AUROC. We performed bootstrapping with replacement and performed 1000 iterations to estimate the 95% CIs for these metrics.

### Evaluation of Llama 3.2 for Zero-Shot NID Classification

We assessed the performance of the Llama 3.2 auto-regressive model (3B parameters) as a zero-shot learning approach for classifying NID from clinical reports in the MGB test set. Llama was neither fine-tuned nor trained on task-specific examples and classified the presence or absence of NID from clinical notes formatted in JavaScript Object Notation.

## Results

### Cohort Characteristics

The demographic characteristics of the 2469 patients comprising the 3000 MGB clinical notes are presented in Table 1 (the linkage between notes and demographic data for the BIDMC dataset was lost and is therefore not shown).

**Table 1.** Characteristics of the study cohorts.

MGB (N=2469)	
Age (years)	
Median (IQR)	61.0 (46-73)
Age group, n (%)	
<18	101 (4.1%)
18‐34	254 (10.3%)
35‐49	403 (16.3%)
50‐64	718 (29.1%)
65+	993 (40.2%)
Sex, n (%)	
Male	1119 (45.3%)
Female	1350 (54.7%)
Race, n (%)	
White	1911 (77.4%)
Black	174 (7.0%)
Asian	86 (3.5%)
Other	298 (12.1%)
Ethnicity, n (%)	
Hispanic	252 (10.2%)
Not Hispanic	2041 (82.7%)
Missing	176 (7.1%)

The median age (IQR) was 61.0 (46‐73 y) years, of which 55% (n=1350) were female sex at birth, 77% (n=1911) reported being of white race, and 83% (n=2041) reported being of non-Hispanic ethnicity. Out of the 3000 notes, 16% (479 notes) were labeled as NID based on expert review, and 97% of these 479 notes had an NID-related ICD code. Among the 1500 notes with an NID-related ICD code, 465 notes (31%) were confirmed as NID cases by expert review. Conversely, among the 1500 notes without an NID-related ICD code, 0.93% (14 notes) were identified as NID cases by expert review. The sensitivity of detecting NID patients using the presence of any NID-related ICD code was 97.1%, and the specificity was 59.1%, suggesting reliance on ICD codes alone for a patient’s true clinical diagnosis may be more likely to overestimate, rather than underestimate, the number of cases with NID.

### Performance of EHR NID Classifier

The training set consisted of 2400 notes from MGB, where 16.1% (387/2400) were classified as NID-positive by expert review. The hold-out testing set from MGB consisted of 445 notes, where 16% (71/445) were classified as NID-positive.

Performance characteristics using XGBoost on the MGB training set and hold-out test set are shown in Table 2.

**Table 2.** Average performance for the XGBoost^a^ model on the training set and 2 independent testing sets.

Metric	Training set (MGB) (2400 notes)	Testing set (MGB) (445 notes)	Testing set (BIDMC^b^) (600 notes)
AUROC^c^, median (95% CI)	1.000 (1.000‐1.000)	0.977 (0.964‐0.988)	0.976 (0.961‐0.989)
AUPRC^d^, median (95% CI)	1.000 (0.999‐1.000)	0.894 (0.831‐0.943)	0.779 (0.655‐0.885)
F1 score, median (95% CI)	0.987 (0.978‐0.995)	0.822 (0.752‐0.879)	0.658 (0.528‐0.778)
Recall, median (95% CI)	0.974 (0.957‐0.989)	0.846 (0.753‐0.923)	0.687 (0.538‐0.839)
Precision, median (95% CI)	1.000 (1.000‐1.000)	0.802 (0.709‐0.889)	0.637 (0.487‐0.795)
Specificity, median (95% CI)	1.000 (1.000‐1.000)	0.960 (0.939‐0.978)	0.976 (0.963‐0.988)

a XGBoost: extreme gradient boosting model.

b BIDMC: Beth Israel Deaconess Medical Center.

c AUROC: area under the receiver operating characteristic curve.

d AUPRC: area under the precision-recall curve.

**Figure 2.**
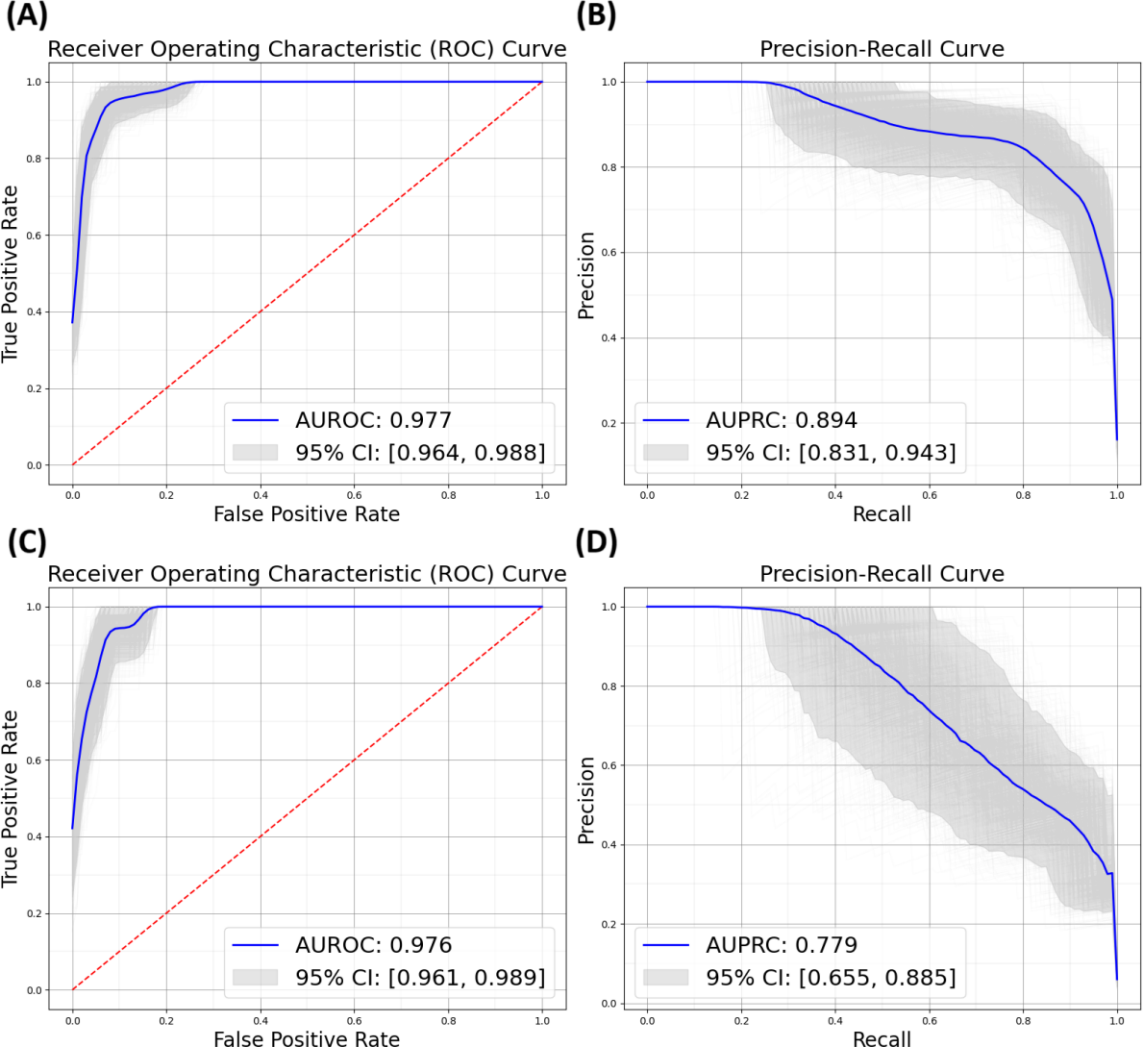
XGBoost model performance on 2 sets of unseen notes, as evaluated by AUROC and AUPRC. 95% CIs were estimated using 1000 bootstrapping iterations. (A) AUROC and (B) AUPRC on notes from MGB and (C) AUROC and (D) AUPRC on notes from BIDMC. AUROC: area under the receiver operating characteristic (ROC) curve; AUPRC: area under the precision recall curve; CI: confidence interval.

The MGB hold-out test set showed good performance metrics (95% CI), including an AUROC of 0.977 (0.964‐0.988), AUPRC of 0.894 (0.831‐0.943), recall of 0.846 (0.753‐0.923), and F1 score of 0.822 (0.752‐0.879). The AUROC and AUPRC curves for the hold-out set are illustrated in Figure 2.

To assess performance metrics using patient notes extracted outside the MGB network, we ran the NLP-based model on 600 notes from BIDMC, of which 5.8% (35/600) were NID-positive by expert review. Although performance declined compared to the MGB test set, the model maintained a high AUROC of 0.976 (95% CI 0.961‐0.989) and an AUPRC of 0.779 (95% CI 0.655‐0.885). Precision was 0.637 (95% CI 0.487‐0.795), and recall was 0.687 (95% CI 0.538‐0.839), resulting in an F1 score of 0.658 (95% CI 0.528‐0.778).

Additionally, we assessed the performance of Llama 3.2, a zero-shot learning model, for classifying NID as present or absent using clinical notes on the MGB test set. The model achieved an AUROC of 0.800 and a balanced F1 score of 0.799, demonstrating high specificity (0.940) but lower recall (0.640). These results highlight notable performance differences between the Llama model and the NLP-based approach, particularly in recall and overall F1 score.

### Significant Features for NID Prediction

**Figure 3.**
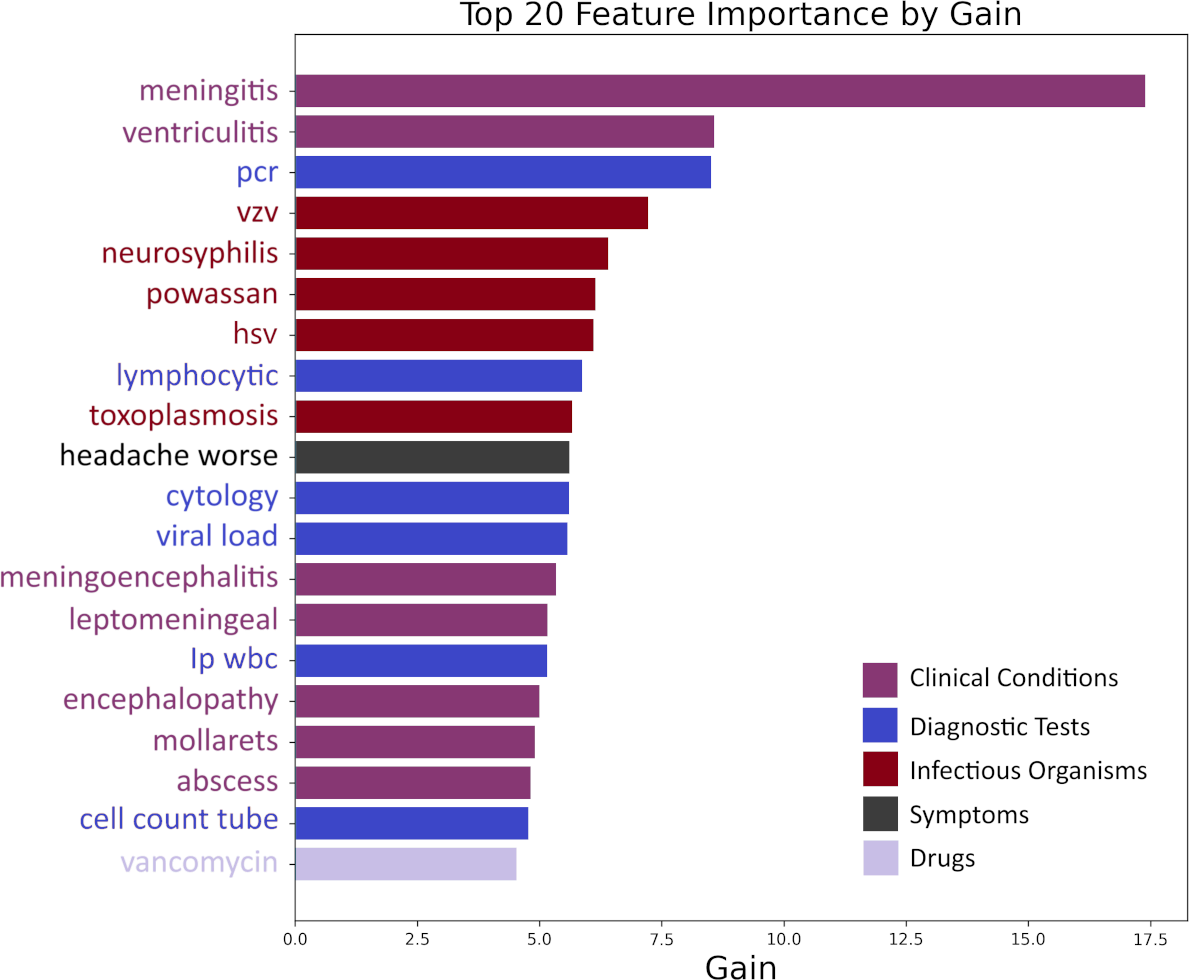
Top 20 features ranked by the feature importance (information gain measured by entropy), arranged in descending order (from top to bottom). The x-axis is the feature importance. It does not differentiate the association directions, ie, positive or negative association with the NID label.

The feature selection steps reduced the initial training feature sets by 73.36% (342/1284). The top 20 features selected by XGBoost to identify individuals with NIDs are plotted in Figure 3.

The importance of these features was assessed based on their average gain across all decision points within the model, providing a measure of their contribution to predictive accuracy. These features primarily consist of clinical indicators and diagnostic markers essential for identifying NIDs. Prominently featured are direct markers of CNS inflammation, such as “meningitis,” “ventriculitis,” and “meningoencephalitis.” Other features included diagnostic tests such as “cytology,” “viral load,” and “polymerase chain reaction”; cell types (“lymphocytic”), specific pathogens, and medical conditions associated with NID were identified, suggesting identified NID-associated features used in the NLP classifier were clinically relevant and interpretable.

### Error Analysis

In our analysis of cases where the model’s predictions differed from the manually assigned labels, we discovered a few common factors that led to incorrect positive and negative classifications across both the internal (MGB) and external (BIDMC) datasets.

A common reason for false positives was the presence of prior NID mentions in patient notes. Another frequent issue was cases where patients were awaiting LP results, leading to a manual negative label, but the notes suggested a possible positive. False positives were also caused by notes indicating symptoms suggestive of NID but lacking confirmatory LP results. Additionally, specific pathogens or conditions, like JC virus neurocysticercosis and neurosyphilis, mentioned in the notes but not confirmed as active NID, added to the error rate. False negatives were often due to notes that did not discuss NID in detail.

The type of clinical note played a significant role; physical therapy and psychiatric notes were less likely to contain detailed clinical information relevant to NID, leading to both false positives and false negatives.

## Discussion

In this study, we developed and evaluated an automated NLP-based model in 2 independent datasets that classifies people with NID using unstructured notes to generate an NID cohort, with the intention that these cohorts could be used for predictions of susceptibility risk and short- and long-term medical outcomes. The NLP model demonstrated high specificity (0.96), outperforming the specificity observed using NID-specific ICD codes (0.59), suggesting that relying on ICD codes alone may overestimate cases of infectious meningitis or encephalitis. These findings align with recent literature reporting low precision (0.58, 95% CI 0.53‐0.63) for the ICD-10 codes associated with herpesviral (herpes simplex) encephalitis, herpesviral (herpes simplex) meningoencephalitis, and herpesviral encephalitis, common causes of NID, in the well-established Danish National Patient Registry [19]. The lack of specificity underscores the risk of false positives when using ICD coding alone, posing challenges for accurately identifying clinical research cohorts, particularly for rare disorders like NID [50,51].

To contextualize our findings, prior studies in meningitis and encephalitis largely focused on structured data, including lab results, imaging, and other EHR data, to classify meningitis etiology or distinguish subtypes [24,31,32,52-58]. In comparison, alternative machine learning models applied to a Brazilian dataset of 22,602 suspected meningitis cases, including J48, Alternating Decision Tree, and Support Vector Machine, demonstrated varying levels of performance. One model, based solely on observable symptoms, achieved an AUROC of 0.869 for differentiating meningitis from non-meningitis cases [31], while a combined ensemble and decision tree method integrating observable symptoms with rapid CSF analysis attained an AUROC of 0.95 in distinguishing bacterial from viral meningitis in a dataset of known infectious meningitis cases [34]. However, the best model for classifying NID cases (sensitivity 97.0%‐97.6%) was at the expense of specificity (24.0%‐43.0%), limiting utility in reducing false positives in clinical decision-making [59]. In the context of HIV and cognitive disorders, machine learning models leveraging clinical data reported AUROCs of 0.83‐0.87, though most relied on structured cohort data, not data in the EHR, with limited validation in independent datasets [60-62]. These comparisons underscore the value of our NLP-based method as a more precise phenotyping tool, offering an improved balance between sensitivity and specificity for identifying NID cases.

We also evaluated LLMs, focusing on the Llama3.2 model applied in the MGB test set. This method yielded an AUROC of 0.800 and a balanced F1 score of 0.799, with high specificity (0.940) but lower recall (0.640). In contrast, the proposed BOW NLP approach achieved balanced results, with an AUROC of 0.977, an AUPRC of 0.894, and an F1 score of 0.822, with high recall (0.846). On the BIDMC validation set, the NLP model achieved an AUROC of 0.98, and while the performance declined (AUPRC reduced from 0.894 (0.831‐0.943) to 0.779 (0.655‐0.885)) likely due to variations in documentation styles, differences in clinical terminology, and institutional biases in diagnosing rare conditions, the model maintained good discriminative ability. These results demonstrate potential for broader application with further fine-tuning, with enhanced precision and interpretability offering significant advantages for NID phenotyping in clinical research.

In our clinical experience, we have observed that unstructured clinical notes serve as a rich source of information crucial for ensuring clinical accuracy and care for NID patients. Here, using only clinical notes, we achieved consistent AUROC exceeding 0.95 across 2 different EHR systems, with a modest decrease in AUPRC, underscoring the importance of developing classifiers that effectively leverage the information embedded within these notes. Compared to Llama, the BOW approach offers advantages such as reduced computational overhead, faster inference, and ease of deployment in settings with predictable patterns and resource limitations. These attributes make it particularly suitable for time-sensitive applications and adaptable to diverse health care settings. Most importantly, the proposed method’s balanced performance, particularly in the context of NID classification, highlights its utility in both clinical research and practice. The model’s reliance on unstructured notes reduces dependency on specific EHR system designs for structured data, broadening its applicability. By identifying clinically interpretable features such as specific markers of CNS inflammation and diagnostic tests, the model enhances both accuracy and clinical relevance, making it a valuable tool for improving care and outcomes for NID patients.

Our study had several limitations. First, the model was developed and validated using unstructured EHR notes from Massachusetts, impacting its relevance in other regions due to regional variations in medical terminology and disease incidence. For example, region-specific diseases like “Powassan,” found predominantly in northeastern states and the Great Lakes region of the United States, were a feature selected in the XGBoost model and may not generalize to other regions. Second, while notes were enriched for NIDs by considering patients who had an LP procedure, the timing of the procedure in relation to the analyzed notes was not considered when developing the model. This approach aimed to enrich the dataset with relevant LP-related language and details from patient notes while avoiding overfitting the model narrowly to notes from a specific temporal context like the post-procedural period. While this approach prevents overfitting to unique details concentrated in the post-LP notes, it could limit broader applicability. Future work could explore incorporating the temporal relationship between the LP and the notes, which may provide additional context for classification performance. Third, lab results from CSF cell counts or microbial assays were not specifically considered in our analysis. The availability of lab data for each patient is inconsistent due to factors such as its assessment at another institution prior to transfer to a tertiary center, safety concerns regarding CSF retrieval, or delays due to logistical considerations. This NLP-based model, operating independently of lab results, resulted in high AUROC and AUPRC, suggesting that in settings where structured lab data is not available, the tool could potentially generate an NID cohort. Our study focused on broad NID classification and did not subtype into encephalitis or meningitis. We anticipate that future model development will involve training on unstructured EHR notes from diverse sources across the United States and may explore incorporating microbial data and antimicrobial therapy details as features.

## Supplementary material

10.2196/63157 Multimedia Appendix 1 List of *ICD-9* and *ICD-10* codes for NID.

10.2196/63157 Multimedia Appendix 2 List of positive expressions.

10.2196/63157 Multimedia Appendix 3 List of negative expressions.

10.2196/63157 Multimedia Appendix 4 List of antimicrobial expressions.

10.2196/63157 Multimedia Appendix 5 List of stopwords.

## Abbreviation

AD: Alzheimer disease
AUPRC: area under the precision-recall curve
AUROC: area under the receiver operating curve
BIDMC: Beth Israel Deaconess Medical Center
BOW: bag-of-words
CNS: central nervous system
CSF: cerebrospinal fluid
EHR: electronic health record
ICD: International Classification of Diseases
LLM: large language model
LP: lumbar punctures
MGB: Mass General Brigham
NID: neuroinfectious diseases
NLP: natural language processing
